# Thromboembolic Events Are Independently Associated with Liver Stiffness in Patients with Fontan Circulation

**DOI:** 10.3390/jcm9020418

**Published:** 2020-02-04

**Authors:** Tarek Alsaied, Mathias Possner, Adam M. Lubert, Andrew T. Trout, Janvi P. Gandhi, BreAnn Garr, Joseph S. Palumbo, Joseph J. Palermo, Angela Lorts, Gruschen R. Veldtman, Stuart L. Goldstein, Alexander Opotowsky, Jonathan R. Dillman

**Affiliations:** 1 Cincinnati Children’s Hospital Heart Institute, Department of Pediatrics, University of Cincinnati College of Medicine Cincinnati, Cincinnati, OH 45229, USA; Possner.mathias@gmail.com (M.P.); Adam.lubert@cchmc.org (A.M.L.); angela.lorts@cchmc.org (A.L.); alexander.opotowsky@cchmc.org (A.O.); 2 Department of Radiology, University of Cincinnati, College of Medicine Cincinnati, Cincinnati, OH 45229, USA; Andrew.trout@cchmc.org (A.T.T.); jonathan.dillman@cchmc.org (J.R.D.); 3 Summer Undergraduate Research Fellowship, Cincinnati Children’s Hospital Medical Center, Cincinnati, OH 45229, USA; janvi.gandhi@cchmc.org; 4 Division of Pharmacy, Heart Institute, Cincinnati Children’s Hospital Medical Center, Cincinnati, OH 45229, USA; breann.garr@cchmc.org; 5 Cincinnati Children’s Hospital Medical Center, Division of Hematology, Department of Pediatrics, University of Cincinnati College of Medicine Cincinnati, Cincinnati, OH 45229, USA; joseph.palumbo@cchmc.org; 6 Cincinnati Children’s Hospital Medical Center, Division of Gastroenterology, Department of Pediatrics, University of Cincinnati College of Medicine Cincinnati, OH 45229, USA; joseph.palermo@cchmc.org; 7 Adult Congenital Heart Disease, Cincinnati Children’s Hospital and Heart Centre and King Faisal Specialist Hospital and Research Centre, Riyadh 45229, Saudi Arabia; gruschen@me.com; 8 Division of Nephrology, Cincinnati Children’s Hospital Medical Centre, Cincinnati, OH 45229, USA; Stuart.goldstein@cchmc.org

**Keywords:** Fontan operation, single ventricle congenital heart disease, coagulation, thromboembolism, anticoagulation, liver stiffness

## Abstract

**Background:** Thromboembolism (TE) and Fontan-associated liver disease (FALD) are common and lead to significant morbidity in Fontan circulations. Risk factors for TE and the potential link between TE and FALD are not well understood. The objective of this study was to evaluate the association between TE and the severity of FALD based on radiologic liver stiffness. **Methods:** Using a retrospective cohort study design, 85 Fontan patients (aged 27.7 ± 8.2 years) who had liver stiffness measurement were included. Multivariable logistic regression was used to determine independent associations with TE. **Results:** Sixteen patients (19%) had a history of TE after the Fontan procedure at a mean age of 21.4 ± 15.0 years. Patients with TE were significantly older at the time of the last evaluation (33.8 ± 11.7 vs. 26.3 ± 6.5 years, *p* = 0.03). Liver stiffness by MRI and ultrasound was higher in the TE group (5.1 ± 1.4 vs. 4.3 ± 1.2 kPa, *p* = 0.04 and 2.8 ± 0.4 vs. 2.4 ± 0.5 m/s, *p* = 0.04, respectively). On multivariable analysis, higher liver stiffness (odds ratio (OR): 2.12, *p* = 0.03) and older age (OR: 1.11, *p* = 0.03) were associated with TE. **Conclusions:** This study found an association between TE, age, and radiologic liver stiffness.

## 1. Introduction

The Fontan procedure compels systemic venous return to traverse the pulmonary circulation without the benefit of a subpulmonary ventricle [1]. Though this procedure has dramatically increased the survival of those with single ventricle physiology, it is associated with chronically elevated systemic venous pressure and impaired cardiac output augmentation [2]. Fontan-associated liver disease (FALD) is being recognized as one of the major noncardiac complications of Fontan circulation and may be associated with substantial morbidity [3,4]. The etiology of FALD is not entirely clear and is likely multifactorial, with elevated central venous pressure (hepatic afterload), possibly abnormal/obstructed liver lymphatic drainage, as well as serial ischemic liver insults as potential causes [5,6]. Recent studies showed that variable liver fibrosis is a universal finding in all patients with Fontan circulation, with some patients having severe fibrosis [7]. Hepatic fibrosis can be studied using tissue biopsies and non-invasively based on imaging appearance and stiffness of the liver using ultrasound shear wave elastography (SWE) or magnetic resonance elastography (MRE) [8,9]. 

Increased risk of thrombosis is well documented in Fontan patients, and antiplatelet treatment or anticoagulation is recommended [10,11]. Recent evidence suggests that prothrombotic tendencies are associated with hepatic fibrogenesis and sinusoidal obstruction [12,13]. Dhar et al. recently showed that the prothrombotic state, as represented by increased thrombin and factor Xa, is associated with liver fibrosis in patients with a structurally normal heart [12]. The potential link between the hypercoagulable state in Fontan patients and FALD has not been well studied. Understanding this link will give insights into the etiology of FALD, may lead to more thoughtful individualized approach to anticoagulation in this patient population, and could potentially help alter the natural history and severity of FALD.

The objective of this study was to evaluate the association between thromboembolic events as a surrogate and consequence of the hypercoagulable state in Fontan patients, as well as the severity of FALD based on radiologic liver stiffness. Our hypothesis is that thromboembolic events are independently associated with increased liver stiffness in Fontan patients. 

## 2. Experimental Section

### 2.1. Patients

This retrospective study was approved by our institutional review board and was conducted in compliance with the Health Insurance Portability and Accountability Act (HIPAA). The Cincinnati Children’s Hospital Medical Center Committee on Clinical Investigation approved this retrospective study and waived the requirement for informed consent. Institutional electronic medical records were searched (using Insight; Softek Illuminate, Overland Park, KS, USA) to identify all adult patients with Fontan circulation (≥18 years of age) who had undergone MRE or ultrasound with SWE of the liver between January 2012 and December 2018. Patients with heart transplant or Fontan take down before the index liver imaging were excluded. Liver imaging is used as a routine clinical surveillance test for all Fontan patients at our institution with many adolescent and adult Fontan patients getting a liver ultrasound SWE or MRE examination every 1–3 years. When more than one ultrasound SWE or MRE was performed only the most recent test was used for our investigation. Clinical findings, laboratory measures, liver imaging, and cardiac testing (including echocardiogram, exercise test, catheterization, and echocardiograms) were compared between the group with thromboembolic events (TE) and the group without events. 

### 2.2. Clinical Data

Clinical and demographic data, including underlying anatomic diagnoses and type of single ventricle congenital heart disease, were abstracted from the medical record (Epic Medical Systems Corporation, Verona, WI, USA). The type of Fontan was classified as lateral tunnel (LT), extracardiac conduit (ECC), or atriopulmonary (AP) connection. Additional parameters included date of birth, sex, age at Fontan operation, and history of arrhythmia. The history of thromboembolism post Fontan procedure was extracted from the chart, and the timing, location, and presentation were recorded. Thrombotic events were defined as any thrombotic event post Fontan that was discovered due to clinical presentation or during routine imaging. All patients were started on aspirin after the Fontan procedure as per our institutional policy. Other documented variables included New York Heart Association functional class and protein losing enteropathy (PLE). Laboratory data included complete blood count and liver function tests. The liver function tests were obtained as part of the comprehensive assessment of the Fontan circulation and are used as routine screening at our institution. Due to the retrospective nature of the study, not all patients were subjected to all the laboratory, catheterization, or imaging procedures. 

### 2.3. Liver MRE 

**Figure 1:**
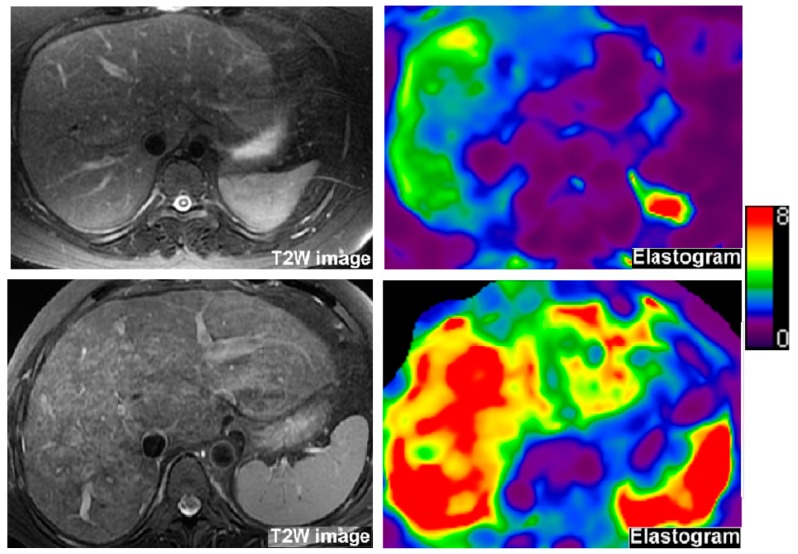
Magnetic resonance elastography images to measure liver stiffness in two patients with history of Fontan palliation. Left images: T2-weighted anatomic images. Right images: MR elastograms showing tissue stiffness. First row of images are from a 31 year-old female with no history of thromboembolism with liver stiffness of 3.0 kPa (upper limit of normal is below 2.5–2.7 kPa). Anatomic images show an enlarged, mildly T2-weighted hyperintense liver compatible with congestion and possible mild fibrosis. The elastogram shows mildly elevated liver stiffness (green and yellow on the color map) with the color scale provided for reference. Second row of images are from a 42-year-old female Fontan patient with liver stiffness of 6.4 kPa. She had atriopulmonary Fontan and a history of bilateral pulmonary embolus due to a right atrial thrombus. Anatomic images show an enlarged liver with subtle surface nodularity and reticular increased T2-weighted signal suggestive of substantial fibrosis in addition to congestion (the hepatic veins appear engorged). There is splenomegaly suggesting portal hypertension. The elastogram shows diffusely increased liver stiffness (e.g., areas of red color).

Liver MRE examinations were performed on 1.5-Tesla MRI scanners (Ingenia; Philips Healthcare, Best, the Netherlands; and Signa HDx or Optima MR450w; GE Healthcare, Waukesha, WI, USA). Both two-dimensional gradient-recalled echo and spin-echo planar imaging techniques were used during the study period, which yield comparable results as shown in previous studies [9,14]. Image acquisition was performed as previously described [15]. Briefly, four axial images were obtained through the mid liver, avoiding the most superior and inferior portions of the liver. Regions of interest (ROI) were drawn on each image to measure shear stiffness, guided by phase and magnitude images. ROIs included the right hepatic lobe as well as segment four of the left lobe, while excluding visible blood vessels, avoiding areas of artifact, and staying at least 1 cm from the liver capsule per our institutional protocol described elsewhere (Figure 1) [15]. Weighted-mean (based on region-of-interest sizes) liver stiffness was abstracted from the electronic medical record by a single investigator. Abdominal imaging was performed to screen for liver neoplasms according to guidelines [16]. 

### 2.4. Ultrasound SWE 

MRE was the primary modality used to assess the liver for our study population, but some patients also underwent abdominal ultrasound with two-dimensional SWE during the follow up period. The ultrasound studies had been performed by dedicated pediatric sonographers who were trained in the performance of shear wave elastography of the liver by the manufacturer at the time of system installation. A total of 10 two-dimensional shear wave speed measurements were obtained in the central right hepatic lobe (or left lobe in the setting of heterotaxy syndrome), at least 1 cm deep to the liver capsule, and away from large vessels. Details of our protocol have been previously published [17].

### 2.5. Cardiac MRI

Cardiac MRI studies had been performed with 1.5-Tesla scanners (Ingenia; Philips Healthcare, Best, Netherlands) and are used for routine screening at our institution. Ventricular assessment was performed using an electrocardiographically gated, balanced, steady-state free precession cine pulse sequence in vertical and horizontal ventricular long-axis planes, and a stack of slices in a ventricular short-axis plane or axial plane encompassing the cardiac apex through the atria. All analyses were performed using commercially available software (QMass, Medis Medical Imaging Systems, Leiden, Netherlands).

### 2.6. Cardiac Catheterization

Cardiac catheterization data included Fontan pathway pressure, ventricular end diastolic pressures, aortic saturation, and pulmonary vascular resistance. Cardiac catheterization was performed according to our standard institutional clinical protocol. 

### 2.7. Cardiopulmonary Exercise Testing 

Exercise testing was performed according to our institutional protocol using a calibrated cycle ergometer and a ramp protocol [18]. For the ramp protocol, the test starts with setting an initial work rate based on patient’s BSA with a linear increase every minute to reach peak exercise after ten minutes. Gas exchange at rest, during exercise, and during recovery was analyzed to determine peak VO_2_ [19]. Peak VO2 was indexed to weight [20]. Exercise capacity was measured by percent of predicted oxygen consumption at peak exercise (% predicted VO_2_) [21]. Only patients with maximal exercise stress testing were included. Maximal exercise was defined as respiratory exchange ratio > 1.09 or a heart rate > 80% predicted. 

### 2.8. Statistical Analysis

The Student’s *t*-test (two-sided) or Mann–Whitney U-test was used to compare two groups of continuous parametric or non-parametric variables, respectively. Fisher’s exact test was used to compare categorical variables between groups. To evaluate the associations of the TE group with liver stiffness as well as other covariates, a multivariable stepwise logistic regression model with 0.1 as the significance level for entry and 0.05 as the significance level to remain in the model was constructed. As anticoagulation treatment was often started after TE, anticoagulation strategy was not included in the model. All p-values were two-tailed, and differences and associations were considered significant when *p* < 0.05. Statistical analyses were performed using JMP® (version 12, SAS Institute Inc., Cary, NC, USA).

## 3. Results

A total of 85 adult Fontan patients (≥18 years of age) who were seen at our institution between 2011 and 2018 were studied. The mean age at most recent evaluation was 27.7 ± 8.2 years, and 47 patients (55%) were females (Table 1). The mean time since Fontan surgery was 19.3 ± 5.7 years. All patients had an echocardiogram evaluation. Seventy patients had MRE, 23 patients had ultrasound SWE, 79 patients had laboratory liver testing, 58 patients had a cardiac catheterization, 50 patients had a cardiac MRI, and 73 had a cardiopulmonary exercise stress test.

Sixteen patients (19%) had a history of at least one thromboembolic event after the Fontan procedure. Of these patients, nine (56%) had an embolic stroke, 2 patients (12%) had a right atrial thrombus, 2 patients (12%) had a lower extremity deep venous thrombosis, 2 patients (12%) had an intracardiac thrombus other than right atrial, and 2 patients (12%) had a pulmonary embolus. One patient had two events (a stroke and deep venous thrombosis) at two different occasions. There was one death in the TE group that was not attributed to the thromboembolic event. 

The mean age at the thromboembolic event was 21.4 ± 15.0 years. The mean time between Fontan and thromboembolic event was 11.4 (range: 0–34) years. Four patients (25%) had the event in the first post Fontan year. Table 1 summarizes the demographic and clinical characteristics of patients with and without a thromboembolic event. Patients in the TE group were significantly older at the time of the last evaluation (33.8 ± 11.7 vs. 26.3 ± 6.5 years, *p* = 0.03). In addition, the TE group was more likely to have a history of arrhythmia which was most commonly atrial arrhythmia (69% vs. 28%, *p* = 0.001) and to be receiving anticoagulation therapy at the time of the last evaluation (69% vs. 29%, *p* = 0.004). The TE group had atriopulmonary Fontan operation more frequently although did not reach statistical significance (38% vs. 16%, *p* = 0.06). 

Table 2 summarizes the most recent cardiac imaging, cardiopulmonary exercise testing, and cardiac catheterization evaluation of the patients in the TE and no TE groups. Both groups had similar hemodynamics by cardiac catheterization and similar ventricular ejection fraction and volumes by cardiac MRI. There was no difference in exercise testing measures, including peak VO_2_, % predicted VO_2_, or VE/VCO_2_ slope. Patients with TE were more likely to have moderate or severe atrioventricular valve regurgitation (30% vs. 9%, *p* = 0.03). 

**Figure 2:**
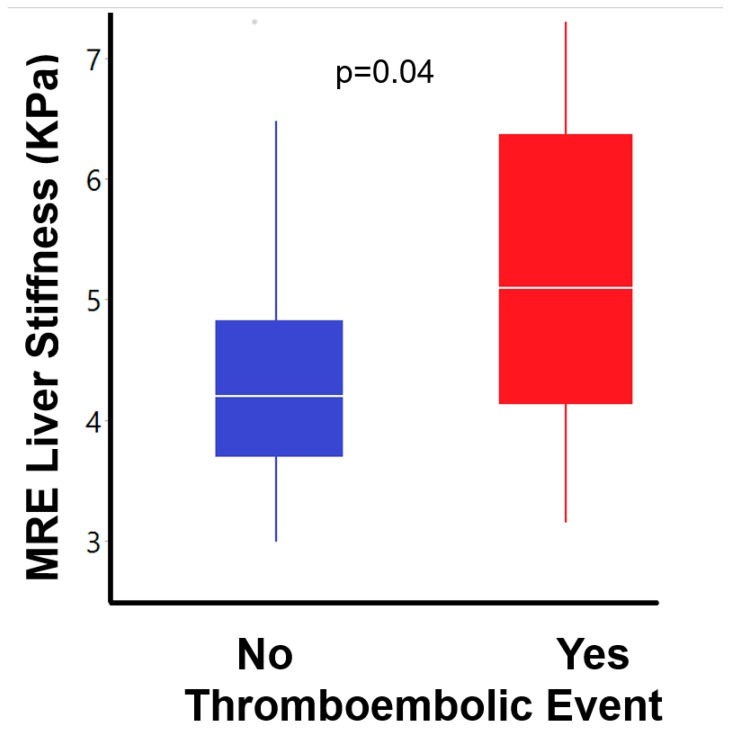
Magnetic resonance elastography shows increased liver stiffness in patients with a history of thromboembolic event represented as a Tukey box plot.

Liver laboratory evaluation showed that patients with TE had higher gamma glutamyl transferase (GGT) levels (142 ± 126 vs. 84 ± 86 units/L, *p* = 0.01, Table 3). Liver stiffness by MRE and ultrasound SWE was higher in the TE group (5.1 ± 1.4 vs. 4.3 ± 1.2 kPa, *p* = 0.04 and 2.8 ± 0.4 vs. 2.4 ± 0.5 m/s, *p* = 0.04, respectively) (Figure 2). Patients with TE were also more likely to have ascites (50% vs. 15%, *p* = 0.01).

On multivariable analysis, only liver stiffness by MRE (Odds ratio (OR): 2.12; confidence interval (CI): 1.08–4.16, *p* = 0.03) and age at most recent evaluation (OR: 1.11, CI: 1.02–1.20, *p* = 0.03) were associated with TE (Table 4). Of note, ultrasound liver stiffness was not included in the model as only 23 patients had ultrasound SWE. 

## 4. Discussion

FALD and TE are important contributors to the long-term morbidity and mortality in Fontan patients. In this study, we reviewed the history of TE in our adult Fontan patients and found independent associations between TE and both age and liver stiffness. 

Despite all the modifications to the Fontan circuit, thrombosis (both arterial and venous) has remained a major complication following the Fontan procedure and increases mortality [10,22,23]. The incidence of TE in our population falls within the range reported in the literature, which varies between 4% and 30% depending on the length of the follow up and the method of detection [10,22,24,25]. Similar to previous studies, patients in the current study presented with intracardiac and extracardiac thrombi, including pulmonary embolism, cerebrovascular thromboembolism, and deep venous thrombosis [10,23,24]. Potential risk factors for thrombosis with Fontan circulation include both hemodynamic factors with low cardiac output and less pulsatile pulmonary flow and well documented abnormalities in coagulation [10,26,27]. Several studies have shown that patients with Fontan circulation carry decreased circulating levels of key anticoagulants, such as protein C and protein S, which could be secondary to FALD or to ongoing consumption [10,26]. Other studies have shown that patients with Fontan circulation have evidence of prothrombotic endothelial activation, including increased circulating levels of von Willebrand’s factor, factor VIII, and soluble thrombomodulin, as well as platelet activation [28,29]. These associations could not be evaluated in our population as testing was not routinely performed on our patients. Our study showed that age was an independent risk factor for thromboembolism. It is well documented in patients with a structurally normal heart that older age is a risk factor of thromboembolism [30]. Increased liver stiffness was also found to be associated with TE in our study. 

Liver stiffness is universally increased in the older Fontan population, initially due to hepatic congestion and eventually due to superimposed fibrosis, and it has been shown to be correlated with worse hemodynamics [15,31,32,33,34]. Recent evidence suggests the added value of repeated monitoring of elastography as a marker of progression of FALD, and the observed association between liver stiffness and TE further supports this [8]. 

**Figure 3:**
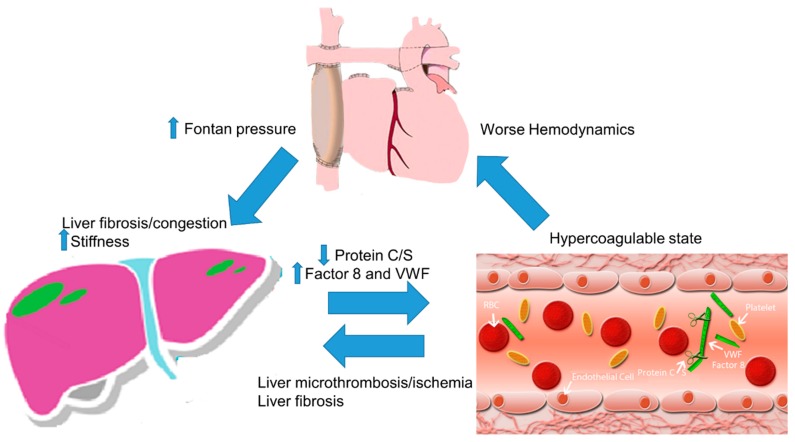
Increased Fontan pressure leads to liver congestion and fibrosis resulting in Fontan-associated liver disease. In turn, Fontan-associated liver disease results in decreased protein C and S and may worsen the hypercoagulable state. The hypercoagulable state may lead to liver microthrombi, activate liver fibrosis, and worsen hemodynamics, which results in a viscous cycle.

While our findings suggest a relationship between TE and worse liver disease measured by increased liver stiffness and increased GGT levels (univariable only), our findings do not explain whether liver disease precedes TE or whether hypercoagulability and TE may lead to worse FALD. FALD was thought to be responsible for at least a portion of the hypercoagulable state seen in Fontan patients [35,36]. Protein C and S levels, which are produced in the liver, are decreased in Fontan patients [10]. On the other hand, TE may result in increased liver fibrosis by two possible mechanisms: (1) TE may cause worse hemodynamics which may lead to worse liver fibrosis and increased liver stiffness [31], (2) recent evidence suggests that hypercoagulable states may lead to increased liver fibrogenesis by inducing microthrombosis and ischemic changes and by directly activating hepatic stellate cell and fibroblasts [37]. Intrahepatic thrombus formation has been shown to play a role in congestive hepatopathy [38] (Figure 3). In a mouse model for congestive hepatopathy and liver fibrosis induced by inferior vena cava ligation, treatment with warfarin was associated with significantly less fibrosis. This supports the hypothesis that thrombosis contribute to liver fibrosis in congestive hepatopathy [38]. This could not be evaluated in our study due to the absence of liver biopsy data. 

A limitation of this study includes its single-center retrospective study design and, therefore, further studies are needed to validate our findings in other Fontan populations. Our study also did not evaluate the impact of the prophylactic antithrombotic strategy on the development of TE and whether that affects the severity of FALD, and we did not routinely measure clotting factors in this population although abnormalities are well documented in the literature. Furthermore, both venous and intracardiac thrombi were included in the analysis as a single group and subgroup analysis could not be performed due to the small sample size. Finally, as mentioned above, due to the retrospective design and the small sample size, our study does not investigate whether FALD increases the risk of TE or whether the hypercoagulable state in Fontan patients contributes to FALD. To investigate causality, future prospective studies will need to look into the progression of liver disease over time and whether it correlates with an increase in the incidence of TE in adolescents and adults with Fontan. 

## 5. Conclusions

This study evaluated the risk factors for TE in a population of Fontan patients and found independent associations between TE and patient age as well as radiologic liver stiffness. Future studies are needed to assess whether anticoagulation strategies could moderate FALD or decrease the risk of FALD progression and its related complications. Furthermore, future studies are also needed to establish if decreasing the severity of FALD lowers the incidence of TE in Fontan patients.

## Figures and Tables

**Table 1:** Demographic and clinical characteristics of the study cohort.

	All Patients (*n* = 85)	Thromboembolic Event		*p* Value
		Yes (*n* = 16)	No (*n* = 69)	
**Age at most recent evaluation (years)**	27.7 ± 8.2	33.8 ± 11.7	26.3 ± 6.5	0.03
**Time since Fontan (years)**	19.3 ± 5.7	22.1 ± 5.8	18.7 ± 5.6	0.08
**Gender (female)**	47 (55%)	9 (56%)	38 (55%)	0.93
**Most recent BMI (kg/m^2^)**	24.2 ± 5.1	27.2 ± 4.4	24.2 ± 5.3	0.86
**Cardiac diagnosis**				0.73
**Tricuspid atresia**	26 (31%)	4 (25%)	22 (32%)	
**Double-inlet left ventricle**	11 (13%)	1 (6%)	10 (15%)	
**HLHS**	21 (25%)	5 (32%)	16 (23%)	
**Unbalanced AV canal**	11 (12%)	1 (6%)	10 (15%)	
**Double-outlet right ventricle**	2 (2%)	1 (6%)	1 (1%)	
**Complex two ventricles**	6 (7%)	2 (13%)	4 (6%)	
**Pulmonary atresia/IVS**	3 (4%)	0 (0%)	3 (4%)	
**Mitral atresia**	3 (4%)	1 (6%)	2 (3%)	
**Ebstein anomaly**	2 (2%)	1 (6%)	1 (1%)	
**Type of Fontan circulation**				0.11
**Atriopulmonary Fontan**	17 (16%)	6 (38%)	11 (16%)	
**Lateral tunnel**	43 (54%)	9 (56%)	34 (49%)	
**Extracardiac conduit**	25 (30%)	1 (6%)	24 (35%)	
**Dominant ventricular morphology**				0.76
**Left ventricle**	50 (59%)	9 (56%)	41 (60%)	
**Right ventricle**	35(41%)	7 (44%)	27 (40%)	
**History of arrhythmia**	31 (36%)	11 (69%)	20 (29%)	0.004
**Protein losing enteropathy**	3 (4%)	1 (6 %)	2 (3%)	0.72
**NYHA class**				0.74
**Class I**	41 (59%)	9 (56%)	40 (57%)	
**Class II**	25 (35%)	5 (31%)	25 (36%)	
**Class III**	4 (6%)	2 (13%)	4 (7%)	
**Anticoagulation at most recent evaluation (warfarin or direct oral anticoagulation)**	30 (35%)	11 (69%)	19 (28%)	0.001
**Aspirin at most recent evaluation**	55 (65%)	5 (31%)	50 (72%)	0.001

AV: atrioventricular, BMI: body mass index, HLHS: hypoplastic left heart syndrome, IVS: intact ventricular septum, NYHA: New York Heart Association, RV: right ventricle, TV: tricuspid valve. Results are presented as mean ± standard deviation or frequency (%).

**Table 2:** Hemodynamic and cardiac imaging results.

	Number of Patients with Each Test	All Patients (*n* = 85)	Thromboembolic Events		*p* Value
			Yes (*n* = 16)	No (*n* = 69)	
**Ejection fraction (%, CMR)**	50	50 ± 8	48 ± 5	51 ± 9	0.14
**End diastolic volume (mL/m^2^, CMR)**	50	98 ± 30	91 ± 14	99 ± 33	0.83
**End systolic volume (mL/m^2^, CMR)**	50	51 ± 24	48 ± 9	52 ± 26	0.56
**At least moderate atrioventricular valve regurgitation (CMR/Echo)**	85	11 (13%)	5 (30%)	6 (9%)	0.03
**Fontan pressure (mm Hg)**	58	13.6 ± 3.9	13.9 ± 2.9	13.5 ± 4.2	0.48
**Ventricular end diastolic pressure (mm Hg)**	58	10.6 ± 3.8	11.1 ± 3.6	10.4 ± 2.9	0.37
**Pulmonary vascular resistance (iWu)**	58	1.5 ± 0.9	1.5 ± 0.6	1.6 ± 0.9	0.93
**Aortic saturation (%)**	58	91 ± 5	91 ± 6	91 ± 5	0.75
**Peak VO_2_ (mL/kg/min)**	73	21.2 ± 6.4	21.1 ± 8.5	22.6 ± 6.0	0.57
**% predicted VO_2_**	73	50.1 ± 15.5	63.5 ± 12.7	59.4 ± 15.9	0.36
**VE/VCO_2_ slope**	73	37.7 ± 7.5	35.9 ± 7.0	38.1 ± 7.6	0.30

CMR: cardiac MRI, iWu: indexed Wood unit. Results are presented as mean ± standard deviation or frequency (%).

**Table 3:** Laboratory and liver imaging results in patients with and without thromboembolic events.

	Number of Patients with Results	All Patients (*n* = 85)	Thromboembolic Events		*p* Value
			Yes (*n* = 16)	No (*n* = 69)	
**MRE liver stiffness (kPa)**	70	4.4 ± 1.0	5.1 ± 1.4	4.3 ± 1.2	0.04
**US liver stiffness (m/s)**	23	2.5 ± 0.5	2.8 ± 0.4	2.4 ± 0.5	0.04
**History of ascites**	85	18 (21%)	8 (50%)	10 (15%)	0.01
**Splenomegaly**	85	19 (23%)	4 (27%)	15 (22%)	0.68
**Portosystemic shunt (varices)**	80	13 (16%)	2 (14%)	11 (17%)	0.82
**Alanine aminotransferase (unit/L)**	79	37 ± 18	43 ± 24	35 ± 16	0.22
**Aspartate aminotransferase (unit/L)**	79	26 ± 10	29 ± 14	25 ± 9	0.37
**Total bilirubin (mg/dL)**	68	0.93 ± 0.65	0.96 ± 0.81	0.93 ± 0.61	0.87
**Gamma glutamyl transferase (unit/L)**	79	100 ± 100	142 ± 126	84 ± 86	0.01
**Total protein (gm/dL)**	79	7.8 ± 0.8	7.7 ± 1.3	7.8 ± 0.7	0.77
**Albumin (gm/dL)**	79	4.3 ± 0.5	4.2 ± 0.7	4.2 ± 0.4	0.91
**Platelet count** **(K/mcL)**	80	191 ± 71	192 ± 73	186 ± 63	0.85

MRE: magnetic resonance elastography, US: ultrasound.

**Table 4:** Univariate and multivariable predictors of thromboembolism.

Predictor	Odds Ratio (95% Confidence Interval) or Parameter Estimate ± SE	*p* Value
Univariate analysis		
Age at most recent evaluation (*n* = 85)	0.09 ± 0.03 ^1^	0.03
Atriopulmonary Fontan (*n* = 85)	3.22 (0.95–10.5) ^2^	0.06
History of arrhythmia (*n* = 85)	5.39 (1.66–17.51) ^2^	0.004
At least moderate atrioventricular valve regurgitation (*n* = 85)	4.83 (1.23–18.89) ^2^	0.03
MRE liver stiffness (*n* = 70)	0.77 ± 0.32 ^1^	0.02
Liver ultrasound SWE (*n* = 23) *	1.50 ± 0.99 ^1^	0.10
History of ascites (*n* = 85)	5.27 (1.63–17.03) ^2^	0.01
Gamma glutamyl transferase (79)	0.004 ± 0.002 ^1^	0.07
Multivariable analysis (*n* = 70)		
Age at most recent evaluation	1.11 (1.02–1.20) ^2^	0.03
MRE liver stiffness	2.12 (1.08–4.16) ^2^	0.03

^1^ Parameter estimate ± standard error. ^2^ Odds ratio for thromboembolism per unit increase. MRE: magnetic resonance elastography. SWE: shear wave elastography. * Not included in the multivariable model due to the small number of patients.
